# An Illustrative Case of Subcutaneous Panniculitis-Like T-Cell Lymphoma

**DOI:** 10.1155/2011/824528

**Published:** 2011-03-03

**Authors:** Farshad Bagheri, Kelly L. Cervellione, Belkis Delgado, Luis Abrante, Jose Cervantes, Jitendra Patel, Alan Roth

**Affiliations:** Departments of Internal Medicine, Clinical Research and Family Practice, Jamaica Hospital Medical Center, 8900 Van Wyck Expressway, Jamaica, NY 11418, USA

## Abstract

Subcutaneous panniculitis-like T-cell lymphoma (SPTCL) is a very rare form of skin lymphoma that is localized primarily to the subcutaneous adipose tissue without palpable involvement of the lymph nodes. Diagnosis of SPTCL is a challenge, especially during its early phases when symptoms mimic other, more common conditions, such as benign panniculitis, eczema, dermatitis, psoriasis and cellulitis. Clinical and systemic features are nonspecific and can include fever, chills, and weight loss. Further complicating diagnosis is the high number of false negatives provided by biopsy. Here we present a case of SPTCL that illustrates the full course of the disease, from presentation and multiple misdiagnoses to correct disease recognition and successful treatment. A review of the challenges of diagnosis is provided with recommendations for more accurate and timely recognition of SPTCL.

## 1. Introduction

Subcutaneous panniculitis-like T-cell lymphoma (SPTCL) is a very rare form of skin lymphoma that is localized primarily to the subcutaneous adipose tissue without palpable involvement of the lymph nodes. It was first described in 1991 in an 8-case series [1] but was not recognized as a distinct entity by the World Health Organization until 2001 [2]. It is estimated that SPTCL accounts for less than 1% of all non-Hodgkins lymphomas [2]. Most often it presents as multiple, painless, subcutaneous nodules on the extremities and trunk. In its early phases, the nodules may resolve without treatment and subsequently new nodules may develop on the same or different skin locations. Diagnosis of SPTCL is a challenge, especially during initial contact with physicians when symptoms mimic other, more common conditions, such as benign panniculitis, eczema, dermatitis, psoriasis, cellulites, and other skin and soft tissue infections. Clinical and systemic symptoms are nonspecific and can include fever, chills, and weight loss; approximately half of patients develop mild cytopenias. More serious conditions associated with SPTCL include hepatosplenomegaly, mucosal ulcers, serosal effusions, hemophagocytosis syndrome (HPS), and pancytopenia, though these are less common [3, 4]. 

Here we describe a case of a middle-aged woman who presented with recurring fevers of unknown origin and an 8-month history of thigh and leg swelling and rash. After several in-patient and out-patient visits, she was diagnosed with SPTCL based on clinical history and results of surgical pathology. Challenges of diagnosis are discussed.

## 2. Case Report

### 2.1. First Admission

**Figure 1:**
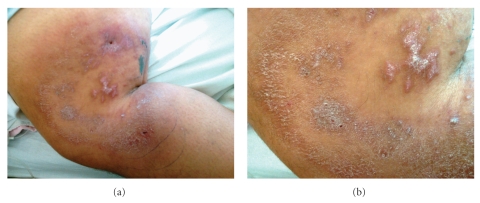
Cutaneous lesions on the thigh, which had been present for 8 months with recent worsening in severity.

A 44-year-old morbidly obese female presented to the emergency department with fever of unknown origin (101.8°F at admission) and chills for 10 days that were unresponsive to antibiotics (cephalexin). She had a history of bulimia, depression, anemia, gastritis, hypertension, and rheumatic fever. She also had chronic skin lesions on the left lower lateral thigh and left upper calf for eight months but reported increased pain in those areas with development of yellow discharge, light bleeding, and numbness over the past 10 days. The rash showed multiple areas of activity and remission presenting as spreading rings (Figure 1). Numerous visits to dermatologists, plastic surgeons, and infectious disease specialists resulted in multiple failed courses of oral antibiotics. Several skin lesion biopsies resulted in differential diagnoses of lichen simplex chronicus versus panniculitis versus prurigo nodularis. A deep fascia biopsy one month prior was interpreted as granulomatous panniculitis with negative stains for acid-fast bacilli and fungi. 

At admission, labs were within normal limits with the exception of low WBC (3.2 K/uL, Normal = 4.8–10.8 K/uL) and abnormal liver function tests (LFT) (BUN = 6 mg/dL, N = 7–17 mg/dL; ALT = 79 U/L, N = 9–52 U/L; AST = 85 U/L, N = 14–36 U/L; AlkPhos = 164 U/L, N = 37–126 U/L). Vancomycin and Zosyn were started. Wound culture was positive for *Streptococcus agalactiae.* Deep tissue biopsy was performed. HIV and Lyme tests were negative. There was progressive worsening of neutropenia (WBC = 2.4 K/uL) and LFT (BUN = 4 mg/dL; ALT = 116 U/L; AST = 146 U/L; AlkPhos = 212 U/L). Hepatitis B and C tests were negative. Fever improved with antibiotics and patient was discharged home on oral levofloxacin. 

Results of biopsy of left upper thigh showed interface/lichenoid dermatitis with granulomatous features and lobular fat necrosis. Left lower leg biopsy showed similar, but more subtle, results. At this time, the pathologist differential diagnoses included collagen vascular disease (e.g., lupus erythematosus), foreign body reaction/unusual drug eruption, sarcoidosis, and Kikuchi's disease. Culture from biopsy grew *Pantoea Agglomerans.* Due to clinical presentation and positive culture results, infectious etiology was suspected. A four-week course of ciprofloxacin was prescribed.

### 2.2. First Admission

Three months later, the patient was readmitted with periodic high fever, chills, nausea, night sweats, and vomiting. She continued to be neutropenic (WBC = 2.9) with slightly elevated LFT (ALT = 57 U/L; AST = 57 U/L; AlkPhos = 149 U/L). Lesions were grossly unresponsive to previous antibiotics. Wound cultures and cancer antigen 125 were normal. After excisional biopsy from the left lower leg and the left groin area, including a lymph node, she was discharged home on antibiotics. The biopsy samples were submitted to an outside lab for review. 

**Figure 2:**
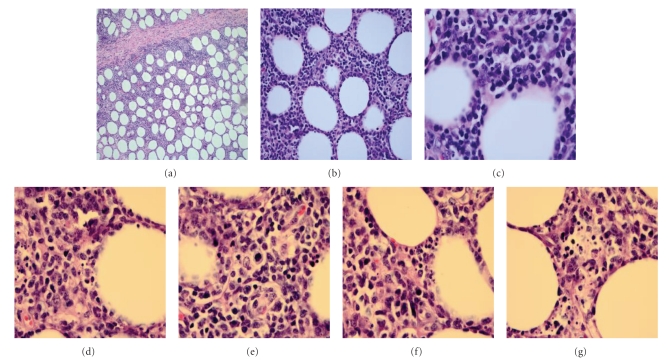
Biopsy of soft tissue from the left thigh revealed: (a) at 10x magnification shows lymphoid infiltrate with pattern resembling lobular and septal panniculitis, and (b) at 40x magnification shows lymphoid infiltrate with abundant cytoplasm. At 100x magnification with oil-immersion biopsy showed (c) atypical lymphoid cells with atypical and irregular nuclei as well as numerous fat cells, (d) numerous single-cell necroses and atypical lymphoid infiltrate, (e) mitotic figure in the center with areas of necrosis and fibrosis, (f) mitotic figure with scattered histiocytes and area of necrosis, and (g) area of necrotic debris.

Surgical pathology for the left lower leg was consistent with mild perivascular small lymphocytic infiltrate without specificity. On low-power field, results for the left groin area sample showed extranodal fibroadipose tissue containing a lymphoid infiltrate with a pattern similar to lobular and septal panniculitis (at 10x magnification) (Figure 2(a)). On high-power field (Figures 2(b)–2(g)), the lymphoid cells were markedly atypical with irregular nuclei. There were numerous fat cells rimmed by atypical lymphoid cells as well as abundant single-cell necrosis. There were scattered and dispersed histiocytes, some with phagocytic cell debris. The septae had areas of necrosis and fibrinoid deposition. The lymph node biopsy revealed a spectrum of small, medium, and some large cells. These results are consistent with SPTCL.

Immunohistochemical stains for the left groin area sample were positive for CD3 and CD8 but negative for CD4 and CD56 (Figure 2). CD4 was positive in scattered small lymphocytes and frequent interspersed histiocytes. CD20 was positive in rare, scattered B-cells. The lymph node had preserved architecture, reactive germinal centers, and paracortical hyperplasia with a spectrum of small, medium, and some large cells; there was no definite involvement by lymphoma. Based on surgical pathology results, a diagnosis of SPTCL involving extranodal adipose tissue in the soft tissue of left groin area was made. The surgical pathology results were most consistent with an SPTCL phenotype referred to as TCR*αβ*. The patient was referred to oncology and began CHOP (i.e., Cyclophosphamide, Adriamycin, Vincristine, and Prednisone) therapy regimen.

### 2.3. First Admission

**Figure 3:**
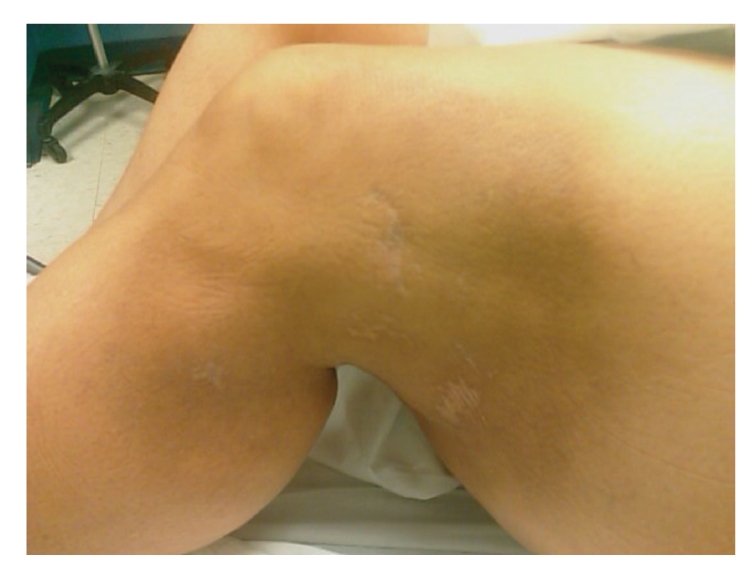
Cutaneous lesions after treatment with five cycles of CHOP chemotherapy (Five months after second admission).

Patient was admitted 5 months later for complicated skin and skin structure infection (CSSSI) of the lower abdomen and mons pubis, right groin, and upper thigh, due to culture-proven MRSA. The areas that had previously showed signs of SPTCL were responding very favorably to CHOP therapy (Figure 3). CSSSI was successfully treated with 4 weeks of IV daptomycin and rifampin. Patient continues to follow up with the oncologist for further treatment, as necessary.

## 3. Discussion

There are two distinct types of SPTCL, classified by the T-cell receptor (TCR) phenotype and immunophenotypic characteristics. The first, TCR*αβ*, is characterized by an indolent, protracted course and is usually CD4^−^, CD8^+^, and CD56^−^. The second, TCR*γδ*, is associated with rapid clinical deterioration and coexisting hemophagocytosis syndrome (HPS); it is usually CD4^−^, CD8^−^, and CD56^+^ [5]. Currently, the medical community uses the SPTCL designation for patients with TCR*αβ*, whereas TCR*γδ* is designated as cutaneous gamma/delta positive T-cell lymphoma (C*γδ*-TCR) [4, 6]. For the purposes of the current report, SPTCL is being used to denote the umbrella of patients with TCR*γδ* or C*γδ*-TCR, while the individual phenotypes are being used to describe the specific phenotypes. 

Diagnosis of SPTCL is based on pathological examination of skin and subcutaneous tissue, immunohistochemical staining patterns, molecular analysis, and clinical characteristics. Patients with SPTCL present with nodules or plaques; ulcerations may also develop, but this occurs in a significantly larger proportion of patients with the C*γδ*-TCL phenotype (45% versus 6% in TCR*αβ*) [4]. B-symptoms (e.g., fever, night sweats, and weight loss) and cytopenias are common in both phenotypes. Approximately 75% of patients with SPTCL have multifocal cutaneous involvement. Lesions typically develop on the arms, legs, and/or trunk [4]. Previous to diagnosis of SPTCL, the patient we report on had numerous visits to both in-patient units and out-patient clinics where benign panniculitis was the primary diagnosis, a common occurrence in cases of SPTCL. 

As described and illustrated by Parveen and Thompson [6] and Paulli and colleagues [7], SPTCL is characterized histologically by neoplastic lymphoid cells infiltrating mainly the lobular areas of the subcutaneous tissue. In cases of C*γδ*-TCL, involvement of the epidermal/dermal tissue may also exist. Lymphocytes exhibit slight atypical features, including hyperchromatic, angulated nuclei, and indistinct cell borders. Admixed benign histiocytes, plasma cells, and neutrophils are present, mimicking benign panniculitis [8]. Scattered mitoses, apoptotic cells, karyorrhectic debris, focal areas of fat necrosis, and rimming of individual fat cells by neoplastic cells are also common in SPTCL [9–11]. 

Histologically, SPTCL can be misinterpreted as other, more common diseases and conditions. Rimming of fat cells can be seen in primary and secondary cutaneous lymphomas and in lobular panniculitis [12]. Some cases of SPTCL have features of vague granuloma formation, which is also present in granulomatous panniculitis. Vasculitis has been rarely reported; angiocentricity and angiodestruction are not features of SPTCL. As in our case, multiple, sequential biopsies may be needed for accurate diagnosis. Use of high power fields is recommended. 

Immunophenotyping is very helpful for diagnosing SPTCL. The neoplastic cells in SPTCL are cytotoxic T cells that are CD3^+^ and CD4^−^. TCR*αβ* cells are CD8^+^ and usually CD30^−^ and CD56^−^ whereas C*γδ*-TCL is usually CD8^−^, CD56^+^, and CD30^+^ [3, 4, 13]. In terms of differential diagnosis, benign panniculitis usually has aggregates of CD20^−^ B-cells mixed with CD3^−^ cells that are both CD4^−^ and CD8^−^ [4]. Lupus erythematosus panniculitis is commonly CD4^+^ without CD8^+^ T cells [14]. Primary cutaneous CD56^+^ natural killer-like T-cell lymphoma is both CD30^+^ and CD56^+^, and unlike SPTCL, is often positive for Epstein-Barr [15]. In our case, the left groin tissue sample exhibited classic immunophenotyping characteristics of TCR*αβ*, including abnormal lymphoid cells from extranodal tissues that were CD3^+^, CD4^−^, CD8^+^, and CD56^−^. Additionally, scattered B-cells were CD20^+^. Taken together, these findings rule-out most differential diagnoses. 

One differential diagnosis that is of particular interest is Pfeifer-Weber-Christian Disease (PWCD), which is a rare inflammatory disorder of the subcutaneous adipose tissue. PWCD is characterized by recurrent subcutaneous inflammatory nodules paired with systemic symptoms, such as fever and malaise. It has been theorized that it is a T-cell mediated autoinflammatory condition [16]. Since PWCD is a diagnosis based on exclusion, and since it can present very similar to SPTCL, it is generally thought that some, if not many, described cases of PWCD may indeed be SPTCL. Furthermore, since cyclosporine is an often-successful form of treatment for PWCD and for TCR*αβ*, misidentification of the disease becomes more of a possibility. 

Imaging technology has begun to be utilized in the diagnostic process; it is also being used for monitoring disease response to therapy. Positron emission tomography (PET) scanning may be useful for staging SPTCL and also for monitoring treatment responses while a patient is undergoing chemotherapy [16]. Whole body MRI is also appropriate for assessing the extent of disease and examining response to treatment [17].

As described by Go and Wester [18], lesions due to TCR*αβ* initially respond well to treatment with steroids, but more than 70% of responders achieve only short remission periods. Further research regarding the use of ciclosporins for treatment of SPTCL is ongoing [19]. Exclusive treatment with radiotherapy in cases of localized skin lesions is highly effective, with reported partial or full response rates of around 80%. CHOP-like therapies are the most commonly used initial types of chemotherapy, with overall complete or partial remission rates of 50%. Few cases have reported use of intensive chemotherapy (e.g., ProMACE-CytaBOM, MACOP-B) or fludarabine-based chemotherapies as initial treatments, although these have been relatively effective with overall remission rates of more than 70%. Numerous types of salvage therapies have been used, though rarely, including ALL, Mini-BEAM, and ESHAP. When implemented, remission rates are lower than for CHOP-like chemotherapy. High dose chemotherapy is the most effective treatment with more than 90% remission, but is very rarely used. In some cases, autologous bone marrow transplants and stem cell transplants have been implemented. Biologic agents, such as interferon-*α*, have been used for a limited number of cases without strong evidence of effectiveness [18].

Treatment of aggressive cases of SPTCL with anthracycline-containing chemotherapy has generally had poor outcomes. There has been one report of successful use of purine analog fludarabine in combination with mitoxantrone and dexamethasone (FND) for treating a multifocal and aggressive CD8^+^ SPTCL [20]; the patient achieved complete remission after one course of treatment and, after a total of six courses, remained in remission for more than 3 years. Additionally, a case of SPTCL with HPS that was resistant to CHOP therapy was reported to achieve complete remission (with 3-year followup) after combination chemotherapy using the BFM-90 protocol [21]. SPTCL in our patient was highly responsive to CHOP therapy. 

The overall five-year survival rate for TCR*αβ* exceeds 80%. The presence of HPS, however, significantly decreases survival rates; the five-year survival rates for TCR*αβ* without HPS are around 90%, whereas if HPS is present the rate falls to less than 50%. On the other hand, there is rapid clinical deterioration in C*γδ*-TCL whether HPS is present or not, with a 5-year survival rate of less than 20% in either group [3, 4]. Even without treatment, metastasis of TCR*αβ* SPTCL is very rare. Metastasis is more common in C*γδ*-TCL, and metastatic sites have included the lungs, liver, kidneys, CNS, and oral mucosa.

 SPTCL is a rare disease that can be a challenge to diagnose. When a patient presents with a long history of panniculitis-like lesions that have been unresponsive to topical and antibiotic therapy and are accompanied by nonspecific clinical symptoms, SPTCL should be considered. Neutropenia that does not respond to antibiotics is another clue for diagnosis. Biopsy with a sample deep enough to include subcutaneous tissue is essential; additionally, high power fields to analyze samples should be used in order to make an accurate differential diagnosis. Immunochemistry should be completed since it is the best way to differentiate between SPTCL and other, more common diagnoses, as well as to guide treatment decisions. Delayed diagnosis can lead to worsening of symptoms and higher probability of morbidity, such as severe skin infections.
